# Multi-marker Similarity Enables Reduced-Reference and Interpretable Image Quality Assessment in Optical Microscopy

**DOI:** 10.34133/research.0783

**Published:** 2025-07-18

**Authors:** Elena Corbetta, Thomas Bocklitz

**Affiliations:** ^1^ Leibniz Institute of Photonic Technology, Member of Leibniz Health Technologies, Member of the Leibniz Centre for Photonics in Infection Research (LPI), 07745 Jena, Germany.; ^2^ Institute of Physical Chemistry (IPC) and Abbe Center of Photonics (ACP), Friedrich Schiller University Jena, Member of the Leibniz Centre for Photonics in Infection Research (LPI), 07743 Jena, Germany.

## Abstract

Optical microscopy contributes to the ever-increasing progress in biological and biomedical studies, as it allows the implementation of minimally invasive experimental pipelines to translate the data of measured samples into valuable knowledge. Within these pipelines, reliable quality assessment must be ensured to validate the generated results. Image quality assessment is often applied with full-reference methods to estimate the similarity between the ground truth and the output images. However, current methods often show poor agreement with visual perception and lead to the generation of various full-reference metrics tailored to specific applications. Additionally, they rely on pixel-wise comparisons, emphasizing local intensity similarity while often overlooking comprehensive and interpretable image quality assessment. To address these issues, we have developed a multi-marker similarity method that compares standard quality markers, such as resolution, signal-to-noise ratio, contrast, and high-frequency components. The method computes a similarity score between the image and the ground truth for each marker and then combines these scores into an overall similarity estimate. This provides a full-reference estimate of image quality while extracting global quality features and detecting experimental artifacts. Multi-marker similarity provides a reliable and interpretable method for image quality assessment and the generation of quality rankings. By focusing on the comparison of quality markers rather than direct image distances, the method enables reduced-reference implementations, where a single field of view is used as a benchmark for multiple measurements. This opens a way for reliable automatic evaluation of big datasets, typical of large biomedical studies, when manually assessing single images and defining the ground truth for each field of view is not feasible.

## Introduction

**Fig. 1.**
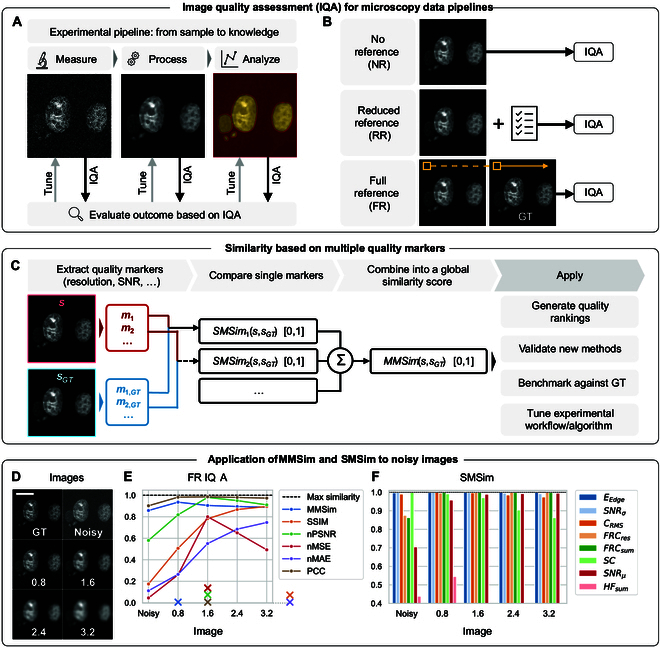
Image quality assessment (IQA) for fluorescence microscopy: introduction to full-reference (FR) metrics and multi-marker similarity (MMSim). (A) Experimental pipeline for fluorescence microscopy: the measurement, processing, and analysis steps are implemented to enhance the image quality and retrieve quantitative information from the measured sample. IQA must be applied at each stage to evaluate the outputs and tune the experimental acquisition, the choice of the processing algorithms, and the related hyperparameters. (B) Different approaches for IQA: from the top, NR metrics provide a direct evaluation without the use of reference data, RR metrics compare the image with a set of known requirements, and FR metrics compare the image with the ground truth (GT), computing a pixel-wise or a sliding-window-wise comparison. (C) Schematics for the computation of MMSim. The method is not based on pixel-wise intensity comparison, but on the extraction of global quality markers from the image data (*s*) and the GT (*s_GT_*). Single markers are compared individually by computing single-marker similarity (SMSim) scores, and then they are averaged into a global MMSim score. (D) Simple example application of MMSim to a denoising problem. GT is the reference image, Noisy is the image with mixed Poisson–Gaussian noise, and the following images are denoised by Gaussian filtering with standard deviation (in pixels) as reported in the labels. (E) MMSim compared with 5 state-of-the-art metrics: structural similarity index (SSIM), peak signal-to-noise ratio (PSNR), mean squared error (MSE), mean absolute error (MAE), and Pearson’s correlation coefficient (PCC). nPSNR, nMSE, and nMAE are normalized to be bound to 0 to 1 for a comparable visualization with the other FR metrics. The colored crosses mark the image that scores the best value for each metric: the maximum value for SSIM and nMAE is reached for higher values not shown in the subfigure (for the full figure, see Fig. S2). (F) SMSim for 8 different quality markers applied to the images. SMSim is plotted from the less-varying score (blue, *SNR_σ_*) to the one with highest variation in the dataset (pink, *HF_sum_*).

The advancement of biological and medical studies is made possible by progress in multidisciplinary fields, including the development of experimental techniques that allow reliable collection of large datasets while minimizing sample degradation and enabling accurate processing and analysis of the collected data. In this direction, robust experimental workflows are essential to ensure repeatable results and a reliable diagnosis. Among the established experimental techniques for biomedical applications, optical microscopy includes a family of methods that allow minimally invasive measurements. Optical microscopy emerged as a robust diagnostic tool thanks to the possibility of implementing reliable experimental pipelines to translate experimental measurement into valuable knowledge. To achieve this goal in image-based studies, researchers must implement reliable pipelines to select a good image from the measurement session, enhance the quality of the image, and extract quantitative knowledge (Fig. 1A). The first step of the pipeline is the acquisition of raw data by utilizing experimental techniques to measure the sample. Then, reconstruction and processing steps are implemented to generate an image from the raw data, if necessary, and improve the image quality by removing experimental artifacts. Finally, the image can be translated into quantitative information by image analysis algorithms. These steps are interconnected, and the success of each process depends on the quality of the output of the previous step. For this reason, image quality assessment (IQA) must be integrated into the experimental pipeline to tune the algorithms and validate each intermediate result before moving to the next task. Current microscopy-based studies can generate hundreds of images, which cannot be inspected manually. Therefore, automatic IQA methods are required within every research study for the selection of the good data and FAIR (findable, accessible, interoperable, and reusable) data management [1–5].

Depending on data availability and the knowledge of the experimental process, IQA can be performed choosing from a wide variety of approaches. Irrespective of the implementation, IQA should be objective, reliable, automatized, and interpretable. IQA methods are commonly classified into 3 categories, depending on the knowledge necessary for their implementation: no-reference (NR), reduced-reference (RR), and full-reference (FR) IQA. These 3 approaches require, respectively, no prior high-quality data for comparison, limited knowledge about the characteristics of the ideal image, or the definition of the ground truth (GT), which is used for comparison (Fig. 1B) [6–8]. This paper targets FR IQA methods and provides specific strategies to reduce the amount of required information and move to an RR implementation. Despite the high amount of prior knowledge required, FR IQA is a powerful method to validate experimental techniques, to benchmark new algorithms, and to implement supervised processes. In this context, FR IQA can be implemented with known datasets, for which the GT can be easily defined. However, FR IQA is less feasible in real case scenarios where the output of a measurement is not known a priori. For this reason, it is important to explore RR IQA, where possible [9,10].

FR IQA is based on the definition of some sort of similarity or distance between the GT and the image to be analyzed. In the current state of the art, many FR metrics have been adapted from natural image studies and applied to optical microscopy studies. A widely used similarity metric is the structural similarity index (SSIM) [6], which ranges from −1 to 1 and is maximized for perfect similarity between 2 images. SSIM is composed of 3 factors, which compute the local similarity between 2 images in terms of luminance, contrast, and structure. Other FR metrics define a mathematical distance between 2 images: they include the peak signal-to-noise ratio (PSNR), the mean squared error (MSE), and the mean absolute error (MAE). A third category of FR metrics is represented by correlation measures, such as Pearson’s correlation coefficient (PCC) [11,12].

All of the mentioned FR metrics rely on intensity comparisons computed between single pixels or small sliding windows. In many cases, this local approach results in poor agreement with visual perception and difficult interpretation of the final score. This was underlined by previous studies on perceptual image resolution, which also highlighted that individual FR metrics show a different behavior for different image degradations and are more sensitive to specific image artifacts [13,14]. Many alternative implementations of state-of-the-art metrics have been proposed to address these drawbacks in specific case studies. For example, different replacements have been implemented for SSIM. Multi-scale SSIM has been proposed to take into account the different scales of imaged structures [15]; other variants have been implemented to address the poor agreement of SSIM with visual perception in the presence of blurred and noisy images [16] and to adapt it to the different dynamic range, noise content, and offset of microscopy images compared to those of natural images [17]. For example, a different offset of a high-quality image compared to the GT will result in a strong change in luminance and an increased distance in intensity, leading to a lower quality score regardless of the actual quality and similarity content. All of the issues mentioned above result in a lack of interpretability and reliability of state-of-the-art metrics in some applications and leaves a desire for a more comprehensive IQA [3,8,13,14,18].

To tackle this challenge, we introduce the multi-marker similarity (MMSim), a new approach for FR IQA that generates a similarity score from multiple quality markers extracted from the images and from the GT. As schematized in Fig. 1C, the workflow for MMSim starts with the computation of multiple NR quality metrics for the image under analysis and the GT, such as resolution, signal-to-noise ratio (SNR), contrast, and structural complexity [19]. Then, a single-marker similarity score (SMSim, in the figure) is generated for each marker. Finally, the single-marker similarities are merged into a final overall quality score of the image.

MMSim offers 2 advantages that are particularly important for FAIR data management of large datasets. Firstly, thanks to this implementation, MMSim is interpretable because it can be fragmented into the individual SMSim scores, which give further insights on the origin of the similarity. In addition, we demonstrate that MMSim can be applied with an RR implementation because, being based on the comparison of quality markers rather than pixel-wise similarities, it allows target features to be extracted from a single GT and used as references for multiple measurements. Therefore, MMSim is particularly advantageous when neither a collection of images can be assessed manually nor is a GT available for each field of view (FOV). In this scenario, a single GT can be defined a priori for each measured structure, and the evaluation result can be further interpreted by tracking the behavior of the single markers. MMSim allows the quality comparison of images with different image contents, which has been demonstrated in 4 examples in this paper, ranging from the hyperparameter optimization of denoising algorithms to the determination of the optimal focal position of experimental measurements.

## Results

### SMSim and MMSim working principle

As introduced above, MMSim falls in the category of FR IQA methods and is based on the extraction of multiple quality markers from the images. Here, we demonstrate briefly the working principle of MMSim, following the workflow of Fig. 1C, and the differences with state-of-the-art approaches. The first step in evaluating an image *s* against the ground *s_GT_* is the computation of the quality markers *m_i_*, which are a limited set of NR metrics that have been proved to be reliable markers for the differentiation of a variety of experimental artifacts [19]. Indeed, experimental artifacts generate multiple image degradations that must be detected using a set of parameters. In particular, the default computation of our method includes 8 quality metrics: the Fourier ring correlation (FRC), which provides a resolution estimation and the correlation sum, the SNR, the signal-to-average ratio, the structural complexity, the sum of high-frequency components of the image power spectrum, the edge energy ratio, and the root-mean-square contrast (Eqs. (1) to (6)). These quality markers are already well-known within the optical microscopy community, yet they are most often utilized individually for specific evaluation tasks. In this paper, our goal is to combine them into a single, comprehensive similarity score, which quantifies different aspects of image quality. As we demonstrate later in this paper, this set of metrics can be reduced to optimize the computational time by utilizing only the markers that are relevant to the current image evaluation problem. Once the single markers are extracted by *s* and *s_GT_*, every pair is utilized to generate an SMSim score (Eq. (7)) that is symmetric, bound to 0 and 1, and equal to 1 only if *m_i_* and *m*_*i*,*GT*_ are equal. In the last step, the single-marker similarities are averaged into a global quality score, namely, the MMSim (Eq. (8)). For each result, MMSim is compared with a set of the most common state-of-the-art metrics used by the optical microscopy community, including SSIM, distance-based parameters (MSE, PSNR, and MAE), and correlation-based similarity (PCC) (Eqs. (9) to (13)). More details on the implementation of the metrics and the workflow are reported in Methods.

The bottom panel of Fig. 1 shows an example application of our method to a simple denoising problem. Figure 1D shows a noisy image, the related GT, and a set of denoised versions obtained by applying Gaussian filtering with increasing standard deviation to the noisy image. We applied MMSim and other FR metrics to evaluate which is the best denoised option, as shown in Fig. 1E. The metrics are normalized to have a value of 1 for maximum similarity. The first relevant observation is that the trend of the metrics is not identical, a problem that has been reported frequently in the literature and that underlines the lack of reliable IQA methods. Most of the state-of-the-art metrics assign a higher score to stronger Gaussian filtering, which generates smoothed images with loss of the detailed sample structure. For all state-of-the-art metrics, the smoothest image scores a substantially higher quality than the noisy image. The most smoothed image shows a darker background and noise removal, but also loss of high-frequency features that are beneficial for the interpretation of the content. On the other hand, MMSim assigns a comparable score to the noisy image and the strongly smoothed ones and selects the first denoised version as the best one. From a visual point of view, this choice seems the most reliable for further interpretation of the image content. Figure S2 shows a detailed characterization of this denoising problem, including additional Gaussian filters and the best similarity score for each metric. From Fig. S2, it is obvious that MMSim selects an appropriate degree of smoothing, while all of the other markers select an oversmoothed image. One advantage of MMSim is the possibility of further inspection by plotting the SMSim scores. In Fig. 1F, the SMSim scores are computed for the 8 markers and sorted from the less to the most varying in the dataset: the noisy image differs from the GT especially for high-frequency components, signal-to-average ratio, and FRC; the first Gaussian filter improves these similarities, while an increasing smoothing lowers again the high-frequency similarity and modifies the structural complexity.

### IQA and quality rankings generated by MMSim show good agreement with visual perception

**Fig. 2.**
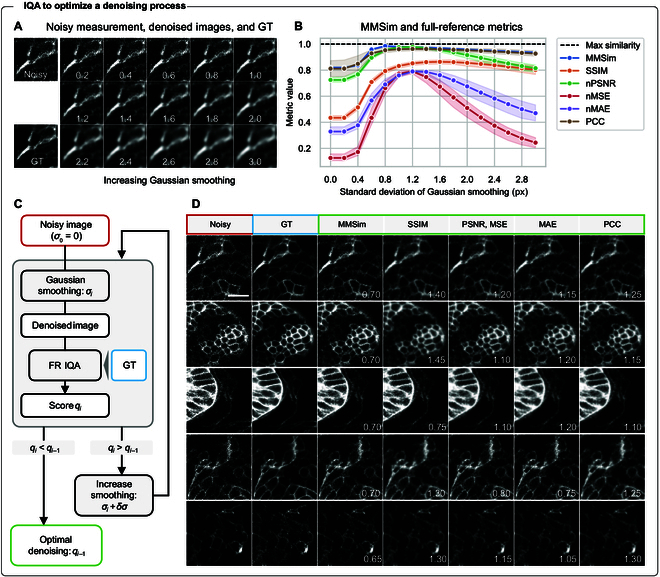
Hyperparameter optimization for denoising with Gaussian filtering. Five noisy measurements of zebra fish embryos are denoised by Gaussian filters with increasing standard deviation. (A) Example cropped images obtained by filtering 1 of the 5 measurements with a Gaussian filter. The image labels indicate the standard deviation of the filter. (B) FR metrics averaged over the 5 measurements. nPSNR, nMSE, and nMAE are normalized to be bound to 0 to 1 for comparable visualization with the other FR metrics. The shaded area is the 95% confidence interval (CI). (C) Schematic of the early stopping algorithm applied to select the best denoised result according to the FR metrics. The process is repeated for every metric, leading to different optimizations. (D) Crops of noisy images, GTs and best images selected by MMSim, SSIM, PSNR, MSE, MAE, and PCC. The white labels indicate the standard deviation, in pixels, of the Gaussian filter applied to each denoised image. The result shows that state-of-the-art metrics, especially SSIM and PCC, are biased toward smoothed images, while MMSim favors higher levels of noise, which usually result in a lower loss of detail. Scale bar: 15 μm.

Here, we further investigate the automatic optimization of a denoising output by FR IQA. First, we start by exploring the behavior of the different metrics for the same filtering problem. Five noisy images of a zebra fish embryo are acquired by fluorescence confocal microscopy, and 5 high-quality images are obtained by averaging 50 independent noisy measurements with the same content [20]. We applied Gaussian filters of increasing standard deviation to the noisy images and used the high-quality images as GT (Fig. 2A). Figure 2B shows the result of MMSim and 5 state-of-the-art metrics averaged over the 5 measurements for each applied filter.

All metrics assign better similarity to smoothed images against the noisy ones and identify the best denoised version within the filtering interval that we selected (i.e., maximum of the curve). PCC, MSE, and MAE show higher variability across the evaluation of different measurements. SSIM is characterized by a maximum shifted toward more smoothed versions, as observed in previous studies [16], while MMSim is characterized by an earlier maximum. This trend can be explained by the fact that state-of-the-art metrics rely on a pixel-wise or window-wise comparison of the images, which is more affected by local intensity variations due to noise and might be improved by smoothing.

Given the different behaviors of the metrics, we can build a simple automatic hyperparameter optimization process to select the best denoised output. Figure 2C shows an early stopping algorithm that takes as input a noisy image and applies iteratively an increasing Gaussian filtering. After filtering, FR IQA is computed for the current metric, and the process continues until the similarity is increasing. Figure 2D shows the optimized images selected by the different FR metrics. This result confirms that MMSim selects images with weaker filtering, while the other metrics, especially SSIM, promote stronger smoothing. While sometimes the amount of noise retained by MMSim seems excessive, it is beneficial for the preservation of details of the measurement, which would be otherwise lost with increasing filtering. Indeed, the MMSim score is a compromise between the generation of good resolution, comparable structural complexity and high-frequency details with the GT, and it is not the result of pixel-wise intensity correspondence.

**Fig. 3.**
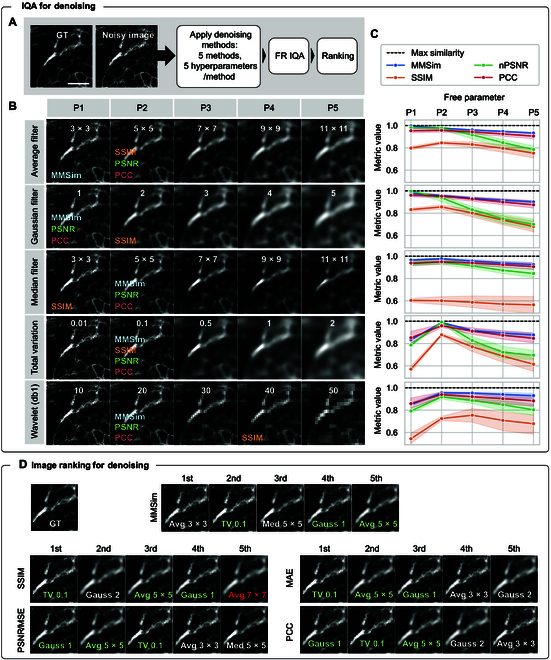
MMSim applied to the IQA and quality ranking of denoised images. (A) Workflow for the generation of the results: a noisy image is corrected by 5 standard denoising methods with a set of 5 parameters for each method. Then, FR IQA is computed, and the quality scores are utilized to rank the images according to their similarity with the GT. (B) Output images processed by 5 denoising methods (rows) and 5 hyperparameters per method (columns). Hyperparameters are reported in each cell, and the name of the metrics indicate the best hyperparameter according to each metric: for example, the 3 × 3 px window is the best average filter for MMSim, while the other metrics select the 5 × 5 px filter. The hyperparameters are window size (px) for the average filter and median filter, standard deviation of the 2-dimensional (2D) Gaussian function for Gaussian filtering, weight for the total variation regularization, and noise standard deviation for wavelet filtering. (C) Trend of 4 FR metrics, averaged on the 5 samples of the dataset, for the varying hyperparameter of each row. Every sample is evaluated with a specific GT. (D) Top 5 denoised images of the field of view shown in panel (B), selected by the FR metrics. Each cell is labeled with the method and hyperparameter (Avg, average filter; Gauss, Gaussian filter; Med, median filter; TV, total variation). Red-labeled images appear only once in the top 5 rankings, whereas green-labeled ones appear in all rankings. Scale bar: 15 μm.

In Fig. 3, we extend this application to hyperparameter optimization of different standard denoising methods and to the generation of image rankings. As schematized in Fig. 3A, we apply 5 denoising methods to the same 5 measurements processed in the previous example. Each denoising method is applied with 5 different hyperparameters, and FR IQA is computed for each output against the available GTs (one for each measurement, obtained by averaging 50 raw measurements). Figure 3B shows the results obtained for 1 of the 5 measurements: the hyperparameters are chosen to provide weakly or almost not denoised images up to images with excessive smoothness and loss of details. Figure 3C shows a subset of FR metrics and MMSim computed for the varying hyperparameters, averaged on the 5 measurements. The metrics show a comparable trend, but there are differences in the selection of the optimized hyperparameter (labeled in Fig. 3B for easier visualization).

On average, MMSim agrees with other metrics and selects images with weaker smoothing, while SSIM is the most biased toward stronger smoothing. SSIM and normalized PSNR show the greatest variability across different samples. On the other hand, MMSim shows a narrow confidence interval (CI). Given that the 5 measurements are acquired under the same experimental conditions and undergo the same process, this behavior is a clue that MMSim correctly evaluates the quality of the image independently of the measured sample. In addition, MMSim shows good agreement with visual perception. Figure S3 shows further inspection of the SMSim and demonstrates that the variability of single markers depends on the specific denoising scenario. Therefore, the selection of a broad range of markers ensures a correct assessment when different approaches are compared.

After the evaluation of the denoised images, an overall quality ranking is generated. Figure 3D shows the top 5 images selected by each FR metrics from the whole dataset of processed images. The labels indicate the applied denoising method and the selected hyperparameter: the labels are white if they occur in at least 2 rankings, green if they are present in all rankings, and red if they occur only in a single ranking. This result confirms that MMSim is the only metric that avoids the selection of excessively smoothed images among the top 5: indeed, in all other rankings, at least 2 images appear blurred and with blunted features, which makes the image interpretation more challenging. Figure S4 shows the same workflow applied to multichannel measurements of bovine pulmonary artery endothelial (BPAE) cells to select the best combination of denoised channels. The assessment shows again that MMSim selects images with sharper features and bright details compared to the state-of-the-art metrics.

### From FR to RR IQA with MMSim

**Fig. 4.**
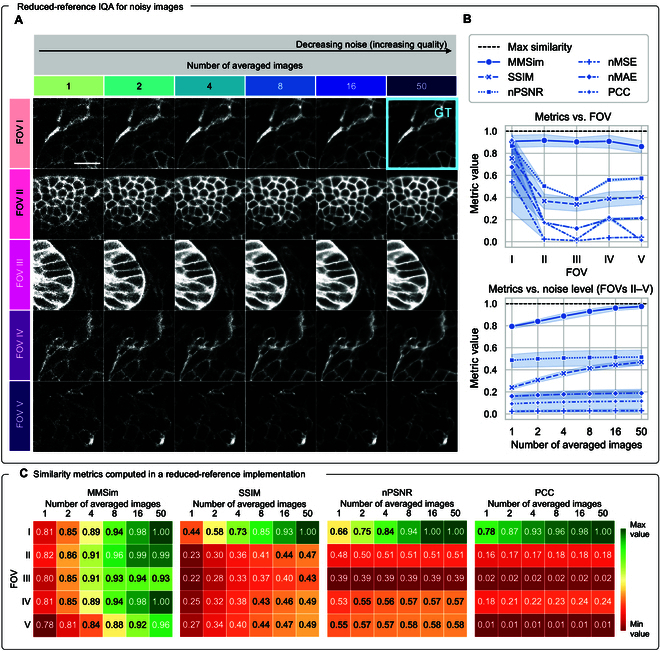
From FR to RR IQA with MMSim: extraction of quality markers independent of the field of view (FOV). (A) Experimental measurements of zebra fish embryos with different levels of noise, obtained by averaging an increasing number of raw images. Each row shows the 6 different levels of noise of a different FOV. The high-quality measurement of the first FOV (light blue frame) is used as single GT for all images. (B) MMSim and other FR metrics computed in RR configuration. Top plot: dependence of the metrics on the FOV, averaged over 6 noise levels. Bottom plot: dependence of the similarity metrics on the noise level, averaged on the nonreference FOVs (II to V). The shaded bar is the 95% CI. (C) Heatmaps showing the trend of 4 similarity metrics computed for the images in panel (A). The color is adjusted in each heatmap to match the full value range, assigning dark green to the best similarity results and dark red to the largest deviations from the GT. The location of the green cells reveals whether high similarity is assigned to high-quality images of different FOVs (as for MMSim) or to different noise levels of the first FOV (as for state-of-the-art metrics). Scale bar: 15 μm.

So far, we have demonstrated the successful applicability of MMSim and some scenarios in which our evaluation method is advantageous compared to state-of-the-art metrics. Now, we can take a step forward and relax some of the constraints of the IQA process. Since MMSim computes the evaluation by utilizing quality markers, it does not work directly on the input images. Therefore, the use of MMSim can be explored in an RR implementation by comparing different measurements of the same sample against the GT from a single FOV. The goal of an RR evaluation is to reduce the amount of prior information (i.e., the knowledge of the GT for every FOV) while keeping an IQA result that depends on the quality of the image and not on the specific measured region. Because the other state-of-the-art metrics are based on pixel-wise and window-wise comparison, this RR approach is not usually feasible. In this example, we use 5 measurements of zebra fish embryos indicated as different FOVs in Fig. 4A. For each FOV, 6 different measurements with decreasing noise are available, which are obtained by averaging an increasing number of raw noisy images. As before, the GT is obtained by averaging 50 raw images, but here we use only the high-quality image of FOV I (light blue frame) as GT for all FOVs and we compute MMSim and the state-of-the-art metrics. The top plot in Fig. 4B shows the metrics against the FOVs, averaged on the 6 noise levels: MMSim is the only metric independent of the FOV, while all other scores show strong variability across different FOVs, which is way higher than the variability generated by the noise levels (represented by the CI). The bottom plot shows the evaluation results averaged on the nonreference FOVs (from II to V) and plotted against the noise level: MMSim is the only metric that evaluates the similarity according to the noise level, while most of the other metrics show a flat curve. SSIM is the only state-of-the-art metric showing a clear increasing behavior with the image quality, affected anyway by a strong offset below the maximum score. These conclusions are confirmed by the heatmaps in Fig. 4C. The cells of the grid are arranged as the images of panel (A), and the colors range from dark green, assigned to the images with the highest similarity to the GT, to dark red, assigned to the images with the lowest similarity. On the left, MMSim shows a color gradient generated mostly by the change in the noise level, with the green images on the right and the red images on the left. SSIM shows a trend that depends on both the noise level and the FOV, while PSNR and PCC are strongly dependent on the image content, with some FOVs featuring the same score across all noise levels.

**Fig. 5.**
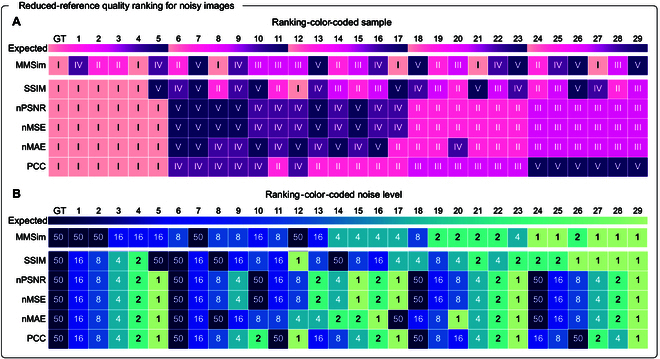
Global quality ranking for measurements of zebra fish embryos (Fig. 4) with increasing noise levels. Each row contains the ranking generated by a different metric in the RR configuration of Fig. 4. The same ranking is represented by assigning a different color to the FOV (panel (A)) and to the noise level (panel (B)). At the top of each panel, a colored bar (Expected) shows the expected color distribution of the images in the ranking: for an ideal evaluation, the result should depend only on the image quality, with shuffled FOVs and a high number of averaged images at the top positions. (A) The color in the ranking indicates the FOV of each image: MMSim ranking is not dependent on the FOV, SSIM ranking is weakly dependent on the FOV, while the other rankings are strongly dependent on the FOV. (B) Same quality ranking as in (A), but the color indicates the number of averaged images, to show whether the ranking depends on the actual image quality. MMSim has a peculiar behavior and generates a quality ranking that is strongly FOV independent and quality dependent, well aligned with the ideal expected color distribution.

Again, we can generate quality rankings of the full dataset from each metric. Figure 5 shows the rankings plotted as heatmaps, where the colors illustrate 2 different pieces of information and correspond to the lateral labels in Fig. 4A: the ranked images are colored based on their FOV in panel (A) and on the number of averaged images (i.e., the noise level) in panel (B). At the top of both panels, we include a colored bar, which represents the distribution of the color-coded images for an optimal evaluation that depends only on the image quality: in an ideal scenario, we expect rankings with shuffled FOVs and a high number of averaged images at the top positions. The rankings confirm that MMSim assigns high similarity to images with low noise levels independently of the FOV, while the other metrics show the opposite behavior. Especially in the 4 bottom rankings, we observe that high-quality images are ranked after noisy measurements. This confirms that among the methods listed here, MMSim is the only approach feasible for an RR evaluation.

The MMSim ranking places 2 high-quality images (average 50, FOV V and III) in positions lower than expected. This behavior can be explained by the fact that these FOVs contain structures different from the GT (see Fig. S1): the bright structures of FOV III and the relatively dark FOV V generate a lower level of similarity than the low-noise images of other FOVs. Figure S5 investigates this behavior using widefield measurements from 20 FOVs of BPAE cell F-actin, demonstrating that MMSim generates the ranking based on the noise level and additional quality features. In Figs. S6 and S7, we demonstrate that MMSim correlates well with the real noise level of the images and, especially for the RR implementation, it outperforms state-of-the-art methods.

### MMSim for RR selection of the optimal focus

**Fig. 6.**
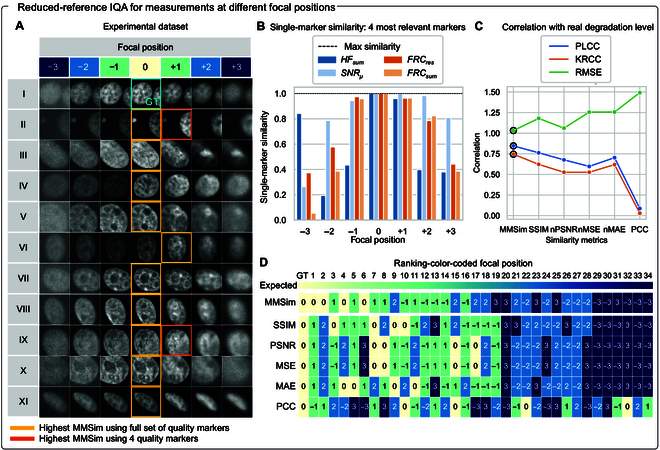
Reduced-reference IQA for out-of-focus images. (A) Eleven measurements of bovine pulmonary artery endothelial (BPAE) cells (rows) at different focal positions along the optical axis (columns). Position 0 is the optimal focal plane; positive and negative numbers indicate measurements above and below the optimal focal plane. Position 0 of FOV I is used as GT (blue frame). The yellow frames indicate the focal position of each FOV with the highest MMSim, computed using the full set of quality markers. The orange frames indicate the highest MMSim computed with the subset of the 4 most relevant markers (panel (B)), if the result differs from the one labeled by the yellow frame. The panel shows 128 × 128 px crops of the original images (full-size images are available in Fig. S1). (B) MMSim for the 4 most relevant markers automatically selected by principal component analysis, computed for FOV I. The GT, located in the center, has the maximum similarity score for all quality markers, while the similarity decreases while moving from the optimal focal plane. (C) Correlation of MMSim and other FR metrics with the real image degradation. Black circles show that MMSim scores the highest Pearson linear correlation coefficient (PLCC) and Kendall rank correlation coefficient (KRCC) and the lowest root mean squared error (RMSE). (D) Global rankings for FOVs I to V generated by different quality metrics (rows). The color indicates the distance from the optimal focus, as indicated in panel (A). The colored top bar shows the expected distribution, dependent only on the image quality: an ideal evaluation result would place the images at optimal focus (0, light yellow) at the top of the ranking, and sort the other images according to the distance from the optimal focus, placing sequentially ±1, ±2, and then ±3 positions.

After having explored many applications of MMSim to denoising problems, we apply our method to the automatic selection of the best focal plane in experimental images of BPAE cell nuclei with an RR implementation. In addition, we demonstrate an automatic approach to reduce the set of SMSim scores to obtain a reliable evaluation of the images while reducing the computational time. Figure 6A shows image crops extracted from the dataset: 11 FOVs are measured at 7 different focal positions (at, above, and below the optimal focal plane). The first FOV is taken as reference, and its optimal focal position is utilized as GT for the whole dataset. The figure highlights with yellow frames the optimal focal planes selected by MMSim for the other 10 FOVs, with perfect agreement with visual perception, and demonstrates the applicability of our method to a defocusing problem.

One drawback of our method is the slower computational time compared to those of other metrics, because MMSim requires the computation of multiple quality markers to generate SMSim scores. In the case of this dataset, the process must be repeated for 70 images (excluding the reference FOV), resulting in a long evaluation process. The set of NR metrics for the computation of the SMSim scores has been selected to provide a comprehensive description of the images and to target a variety of experimental and processing artifacts. However, once the evaluation problem is clearly identified, not all metrics are equally relevant to IQA. Therefore, to reduce the complexity of the problem, we select the 4 most relevant metrics, i.e., the most variable with the defocusing process. The selection of the metrics can be applied after the manual inspection of the SMSim computed for the reference FOV, but also in an automatic fashion, for example, by principal component analysis (PCA) (see Methods for further details). PCA is applied only to the reference FOV to ensure that the variability is not caused by the imaged objects but by the artifact itself. Then, the metrics that contribute the most to the principal components are selected. In our case, the most relevant metrics are high-frequency components, signal-to-average ratio, FRC resolution, and FRC sum (Fig. 6B). By utilizing only this subset, the best focal positions are the same as those selected with the default set of metrics, except for FOV II and FOV IX, as indicated in Fig. 6A by the orange frames. The inspection of the principal components can be also used to automatically tune the weights to combine the single-marker similarities in a unique score, considering which markers contribute the most to the quality variability within the reference FOV.

Figure 6C shows 3 correlation measures between the IQA metrics and the real defocusing level: MMSim shows the best correlation due to maximized Pearson linear correlation coefficient and Kendall rank correlation coefficient and a minimized root mean squared error compared to the other metrics. Exact correlation values are shown in Fig. S8. Figure 6D shows the overall quality ranking generated by all metrics for the first 5 FOVs colored according to the focal position of the measurements. Again, the interpretation of the ranking is supported by a colored bar at the top of the panel, which displays the expected color gradient for an ideal evaluation. MMSim places the optimal focal positions (0) and those close to the optimal focus (+1, −1) at top positions, following the expected trend, while other metrics, especially PCC, sort the image quality also according to the similarity between imaged objects.

## Discussion

In this paper, we have introduced MMSim, an evaluation method for estimating image similarity by comparing multiple image quality markers. The main difference with state-of-the-art FR approaches is the generation of a quality score that is not computed by pixel-wise or window-wise distances between the image and the GT but is based on the extraction and comparison of global quality markers. These markers include, but are not limited to, resolution, contrast, SNR, structural complexity, and high-frequency components.

We compared MMSim with state-of-the-art methods that serve as benchmarks for IQA in optical microscopy: SSIM, PSNR, MSE, MAE, and PCC. Despite some known limitations of these metrics, they are widely adopted due to their proven stability in many evaluation cases and straightforward implementation. Not only does MMSim achieve performance comparable to that of state-of-the-art methods, but it also shows good agreement with visual perception and high stability when applied to images acquired under the same experimental conditions. For example, for denoising applications, MMSim favors images with a low amount of residual noise rather than highly smoothed images, showing an opposite trend compared to state-of-the-art methods and selecting outputs with a higher level of detail. In fact, the indirect evaluation of images through the extraction of global quality markers makes our method strongly dependent on the degradation content of the images and weakly dependent on the specific FOV, as long as the structure of the imaged objects remains similar. For this reason, MMSim offers the advantageous option of implementing an RR evaluation, where a single FOV is used as reference for multiple measurements of the same kind of sample. Moreover, if the target value for a specific marker is known without extracting it from a custom measurement of the biological specimen (e.g., if the resolution of the microscope has been theoretically determined or measured using microspheres), this value can be used to compute the related SMSim. This application provides a more flexible approach than the state of the art.

Thanks to the definition of the quality markers, which are based on physical experimental artifacts and the resulting image degradations, MMSim can be further inspected by looking at the SMSim scores. In this way, the result can be interpreted by revealing which markers affect the similarity with the GT and are relevant for specific applications. Moreover, the use of established quality markers makes our method more reliable and encourages its implementation in biological and clinical studies, where the method’s dependability and stability are most important.

Compared to state-of-the-art FR metrics, the detailed image evaluation and the possibility to gain further insights come at the cost of higher computational time. Moreover, MMSim may fail when applied to image processing tasks that recover the desired quality features by introducing unexpected artifacts that can be identified only by locally inspecting the intensity values of individual pixels. To mitigate the first drawback, we took advantage of the interpretability of MMSim, and we tested an automatic approach for reducing the set of quality markers for specific evaluation problems. To address the second drawback, MMSim could be integrated with state-of-the-art evaluation metrics and used for a complementary IQA. In addition, MMSim could be used as a fine-tuning approach to select among good available options or to interpret the value of other metrics.

In this study, we have demonstrated the successful application of MMSim in a variety of scenarios: the hyperparameter optimization of denoising methods, the generation of large quality rankings in the presence of multiple processing results, the use of an RR implementation to generate quality rankings of noisy images and to optimize the focal position, and the successful simplification of the evaluation process by selecting a reduced set of metrics. Our method has proven to be stable, reliable, and in good agreement with visual perception in all of these cases. A reliable FR IQA approach is essential to validate experimental methods and processing algorithms and to compare images to a known reference. In addition, the ability to interpret the evaluation in terms of individual image features gives further potential for inspecting degradation and processing outcomes to uncover unknown insights during a supervised FR IQA. Furthermore, the same potential can be transferred to an RR implementation, thus reducing the amount of prior information required and making the evaluation feasible for real experimental scenarios. For example, MMSim is feasible for an accurate, automatic, and interpretable IQA for optical microscopy-based biomedical studies. Indeed, MMSim provides IQA, automatic generation of quality rankings, and interpretation of a varied image collection. This is fundamental when assessing the measurement quality of experimental techniques, such as the noise content or blurring, and when applying processing algorithms for image analysis and diagnostic tasks.

## Methods

### SMSim and MMSim

MMSim is based on a straightforward workflow comprising the following steps. Once the GT is defined for a given dataset,

1. A set of NR quality metrics is selected and computed for all images, including the GT. Each metric is considered a quality marker for a specific image property, e.g., resolution, SNR, and contrast.
2. For each quality marker, an SMSim is constructed to evaluate the similarity between the images and the GT according to a single image property.
3. The single-marker similarities are averaged to generate an MMSim that provides an estimate of the global similarity of the images and the GT considering all selected quality markers.

In the following, we provide the definitions for the steps listed above.

#### Single quality markers

The NR metrics are selected to provide a comprehensive IQA of the dataset. They should address the complementary aspects of the images because one single metric is usually not sufficient to detect multiple image degradations caused by experimental and processing algorithms. In a previous study [19], we demonstrated the reliability of a set of quality markers for the characterization of experimental artifacts with NR implementation. The markers were selected after a systematic validation of many established quality metrics and proved to be a robust choice to classify experimental artifacts and generate quality scores. Inspired by this systematic study, we selected 8 NR metrics, whose definition is given below for an image *s*. In the following expressions, *N_pix_* is the number of pixels of the image, *s_max_* is the maximum value of the image, and *μ_s_* and *σ_s_* are the average and the standard deviation of the image or, if available, of a selected background region, respectively. The spatial coordinates are indicated by *x* and *y*, whereas the spatial frequencies are indicated by *f_x_* and *f_y_*.

• FRC. FRC is a correlation-based measure of the spectral SNR of an image, and it is commonly used to quantify the image resolution. FRC is computed by correlating rings of increasing radius extracted from 2 independent versions of the image in the Fourier domain. In this work, we used the single-image implementation of FRC [21] and utilized as markers both the estimated resolution (*FRC_res_*) and the sum of the correlation curve (*FRC_sum_*).
• Signal to noise ratio *SNR_σ_*, defined as the logarithmic ratio between the dynamic range of the signal and the standard deviation of a background region. If the background region is not selected a priori, the full image is used to compute the standard deviation.
  $${\mathrm{SNR}}_{\sigma }\left(s\right)=20\;{\text{log}}_{10}\frac{s_{\max}-\mu_{s}}{\sigma_{s}}$$ (1)
• Contrast to average ratio *SNR_μ_*, defined as the logarithmic ratio between the dynamic range of the signal and the mean value of a background region. If the background region is not selected a priori, the full image is used to compute the mean value.
  $${\mathrm{SNR}}_{\mu }\left(s\right)=20\;{\text{log}}_{10}\frac{s_{\max}-\mu_{s}}{\mu_{s}}$$ (2)
• Sum of high-frequency components *HF_sum_*, defined as the sum of all pixels of the sum-normalized image power spectrum |*F*(*s*)|^2^ lying outside a radial threshold *f_th_*. The radial threshold selects the high-frequency part of the power spectrum, and it is set to three-eighths the full size of the image.
  $${\mathrm{HF}}_{\mathrm{sum}}\left(s\right)=\sum_{f_{x}^{2}+f_{y}^{2}>f_{\mathrm{th}}^{2}}\frac{{\left|F\left(s\right)\right|}^{2}}{\sum_{N_{\mathrm{pix}}}{\left|F\left(s\right)\right|}^{2}}$$ (3)
• Structural complexity *SC*, defined as the average gradient of the image. It accounts for the presence of sharp structures or noise, and its value is lower for images with strong smoothing or with flat intensity profiles.
  $$\mathrm{SC}\left(s\right)=\frac{1}{N_{\mathrm{pix}}}\sum_{N_{\mathrm{pix}}}\sqrt{\frac{\nabla_{x}^{2}\left(s\right)+\nabla_{y}^{2}\left(s\right)}{2}}$$ (4)
• Edge energy ratio *E_edge_*. It is the ratio between the sum of the pixels lying outside a radial threshold *r_th_* and the sum of all pixels of the image. In the presence of vignetting, *E_edge_* is lower. The threshold is set to three-eighths the full size of the image.
  $$E_{\text{edge}}\left(s\right)=\frac{\sum_{x^{2}+y^{2}>r_{\mathrm{th}}^{2}}s\left(x,\;y\right)}{\sum_{x,y}s\left(x,\;y\right)}$$ (5)
• Root-mean-square contrast *C_RMS_*, which provides an additional contrast definition, and it can be affected also by the presence of noise. The image is rescaled between 0 and 1 before the computation of *C_RMS_*.
  $$C_{\mathrm{RMS}}=\sqrt{\frac{1}{N_{\mathrm{pix}}}\sum_{N_{\mathrm{pix}}}{\left(s-\mu_{s}\right)}^{2}}$$ (6)
  The quality markers assume always positive values, and the possible negative values reached for the logarithmic SNRs can be regularized to a small constant *ε*. Prior to the computation of the metrics, the images are rescaled to the interval [0,1]. In this paper, *μ_s_* and *σ_s_* are computed for the full image. Once the markers are computed for the whole dataset, strong outliers that may be generated due to the presence of strong artifacts are removed by locking the minimum and maximum values of the markers to the lower percentile of 0.005 and the upper percentile of 0.995, respectively. Such normalizations are recommended for a large dataset and must be computed consistently for the entire set of images but may be redundant and not necessary for simpler cases.

#### Definition of SMSim

Given an image *s* and the GT *s_GT_*, SMSim is built to quantify their similarity with a quantitative score. SMSim should be symmetric, i.e., *SMSim*(*s*, *s_GT_*) = *SMSim*(*s_GT_*, *s*), and bound to a certain interval independently of the order of magnitude of the quality marker. Various analytical functions satisfy these conditions, and they are characterized by different curves. We have selected for the SMSim an expression that resembles the single factors in the SSIM. The SMSim for a marker *m_i_* is defined as

$${\text{SMSim}}_{i}\left(s,\;s_{\mathrm{GT}}\right)=\frac{2m_{i,s}m_{i,s_{\mathrm{GT}}}+k}{m_{i,s}^{2}+m_{i,s_{\mathrm{GT}}}^{2}+k}$$ (7)

SMSim is 1 if the marker assumes the same value for the 2 images, and it decreases, with a lower limit of 0, if the 2 markers assume different values. A small constant *k* is introduced to regularize the result in the case of very small values of $m_{i,s}$ and $m_{i,s_{\mathrm{GT}}}$, and it is equal to 0.01 the data range of marker *m_i_*. Before the computation of SMSim, the markers are maximum normalized to rescale them to the same order of magnitude. Therefore, the data range of all markers is 1 and *k* = 0.01.

#### Definition of MMSim

MMSim is a global similarity score that accounts for all SMSim scores computed for the images, and it should preserve the bound of the single score. Among the averaging options available, we choose a simple arithmetic average, which provides the same importance to all markers. For a set of SMSim scores *SMSim_i_* computed on *N_m_* markers *m_i_* for an image *s* and the GT *s_GT_*,

$$\text{MMSim}\left(s,\;s_{\mathrm{GT}}\right)=\frac{1}{N_{m}}\sum_{m_{i}}{\text{SMSim}}_{i}\left(s,\;s_{\mathrm{GT}}\right)$$ (8)

MMSim is bound to the interval [0,1], and its value is higher for better similarity of the images.

#### FR state-of-the-art metrics

MMSim is benchmarked to state-of-the-art FR metrics commonly used by the imaging community. In particular, we compare our method with the following:

• SSIM:
  $$\text{SSIM}\left(s,\;s_{\mathrm{GT}}\right)=\frac{\left(2\mu_{s}\mu_{s_{\mathrm{GT}}}+C_{1}\right)\left(2\sigma_{s,s_{\mathrm{GT}}}+C_{2}\right)}{\left(\mu_{s}^{2}+\mu_{s_{\mathrm{GT}}}^{2}+C_{1}\right)\left(\sigma_{s}^{2}+\sigma_{s_{\mathrm{GT}}}^{2}+C_{2}\right)}$$ (9)
  with *C*_1_ = (*k*_1_*L*)^2^ and *C*_2_ = (*k*_2_*L*)^2^, *k*_2_ = 0.01, *k*_2_ = 0.03, and *L* equal to the range value of the image pixels. $\sigma_{s,s_{\mathrm{GT}}}$ is the covariance of the 2 images.
• MSE:
  $$\mathrm{MSE}\left(s,\;s_{\mathrm{GT}}\right)=\frac{1}{N_{\mathrm{pix}}}\sum_{i=1}^{N_{\mathrm{pix}}}{\left(s_{i}-s_{\mathrm{GT},i}\right)}^{2}$$ (10)
• PSNR, defined as
  $$\begin{array}{c} \text{PSNR}\left(s,\;s_{\mathrm{GT}}\right)=20\;{\text{log}}_{10}\frac{s_{\mathrm{GT},\max}}{\sqrt{\mathrm{MSE}\left(s,\;s_{\mathrm{GT}}\right)}} \end{array}$$ (11)
• MAE:
  $$\mathrm{MAE}\left(s,\;s_{\mathrm{GT}}\right)=\frac{1}{N_{\mathrm{pix}}}\sum_{i=1}^{N_{\mathrm{pix}}}\left|s_{i}-s_{\mathrm{GT},i}\right|$$ (12)
• PCC:
  $$\mathrm{PCC}\left(s,\;s_{\mathrm{GT}}\right)=\frac{\sum_{i=1}^{N_{\mathrm{pix}}}\left(s-\bar{s}\right)\left(s_{\mathrm{GT}}-\bar{s_{\mathrm{GT}}}\right)}{\sqrt{\sum_{i=1}^{N_{\mathrm{pix}}}{\left(s-\bar{s}\right)}^{2}}\;\sqrt{\sum_{i=1}^{N_{\mathrm{pix}}}{\left(s_{\mathrm{GT}}-\bar{s_{\mathrm{GT}}}\right)}^{2}}}$$ (13)

#### Workflow for IQA and quality ranking

IQA is executed by computing MMSim and additional FR metrics on the entire dataset, upon selection of the GT. The quality rankings are generated by sorting the images from the highest to the lowest score for each metric. The scores are also computed for the GT itself, which is placed at the top of the ranking with a value of 1. Visual comparison of the metrics can be facilitated by plotting the results. This approach requires the scores to be constrained to the same interval and follow the same trend (increasing or decreasing) for an improving similarity. We decided to standardize all metrics to have an increasing score up to 1 for an improving similarity. MMSim, SSIM, and PCC already satisfy these requirements. PSNR already shows the desired behavior but assumes an infinite value for perfect similarity. Therefore, the infinite value is set to 110% of the highest score of the dataset. Then, percentile normalization is applied with a minimum quantile of 0.05 and a maximum quantile of 0.95 to avoid strong outliers for visualization. MSE and MAE show a decreasing behavior with increasing similarity and have a value of 0 for perfect similarity. Therefore, their value is first clipped between 0.05 and 0.95 quantiles, and then their trend is inverted. For the metric *x* with a value of 0 for perfect similarity, the inverted trend *x_inv_* is obtained by calculating *x_inv_* = *x_min_*/*x*; here, *x_min_* is the minimum value in the dataset. Normalized values of PSNR, MSE, and MAE are indicated in this paper respectively as nPSNR, nMSE, and nMAE.

#### Reduction of the set of quality markers by PCA

To reduce the computational and time effort for specific applications, we integrated a few additional steps in the evaluation workflow. This approach is particularly feasible for RR IQA and for identifying which metrics are strongly affected by image degradations. Initially, the GT is selected among a subset of images of the same FOV, i.e., images with different levels of quality but the same content. Then, the full set of quality markers is computed for these images, and the markers are used to fit a PCA model before any estimation of similarity. Then, the PCA components are inspected by extracting their coefficients and explained variance. The *N* most relevant metrics are selected by rescaling their corresponding coefficients according to the explained variance ratio and summing the resulting values over the set of components. In this paper, we selected the 4 markers with the highest relevance. Finally, the selected metrics are used for the computation of single-marker similarities and MMSim for the entire dataset, including more FOVs and implementing an RR evaluation. This process can be adapted by selecting the number of relevant metrics to be extracted or a threshold for their relevance.

### Datasets

#### Experimental images with different noise levels: Fluorescence microscopy denoising dataset

We utilized confocal and widefield microscopy measurements of fixed BPAE cells and fixed zebra fish embryos from the fluorescence microscopy denoising (FMD) dataset [20]. The confocal measurements of BPAE cells and zebra fish embryos are acquired by a laser scanning confocal microscope with a water immersion objective, a pixel size of 300 nm, and parallel detectors for multichannel measurements. The widefield measurements of BPAE cells have a pixel size of 170 nm and are measured by a single charge-coupled device detector. The measurements are provided with 6 noise levels, obtained by averaging an increasing number of raw measurements. The noisy images are generated by averaging 1, 2, 4, 8, and 16 raw images, while the high-quality images are obtained from the average of 50 raw images. The measurements of BPAE cells are composed by 3 channels: nuclei labeled with 4′,6-diamidino-2-phenylindole (blue), F-actin labeled with Alexa Fluor 488 phalloidin (green), and mitochondria labeled with MitoTracker Red CMXRos (red). The measurements of zebra fish at 2 d post fertilization (EGFP labeled *Tg*(*sox10:megfp*) zebra fish embryo) are composed by a single channel. These experimental images are utilized for evaluating the similarity with increasing levels of noise (Figs. 4 and 5) and for denoising applications (Figs. 2 and 3).

#### Hyperparameter optimization for denoising by Gaussian filtering

The study on the optimization of Gaussian filtering is implemented by iteratively smoothing the image with a bidimensional Gaussian function of increasing standard deviation *σ*, as reported in Fig. 2C. The iterative process is repeated for each FR metric. The standard deviation of the first iteration is set to *σ*_1_ = 0.05 px, and then it is increased at each iteration by *δσ* = 0.05 px. At each iteration, the value of the FR metric is checked, and the process is continued until the value is improved. If an optimization is not obtained within 100 iterations, the process is stopped. The improvement is checked by monitoring the relative increase (for MMSim, SSIM, and PSNR) or decrease (for MSE and MAE) between 2 consecutive iterations, with a tolerance of 1e−5 of the value assumed at the previous iteration, to avoid early stopping due to small oscillations of the metrics.

#### IQA and quality ranking of denoised images

The study on different denoising methods utilizes raw images of the FMD datasets denoised with the following methods: average filter, median filter (SciPy implementation in Python), Gaussian filter, total variation regularization (scikit-image implementation in Python), and wavelet filtering (scikit-image implementation in Python). The GT is selected among the high-quality images obtained by averaging 50 raw images. The hyperparameters utilized for the generation of the denoised images of Fig. 3B are included in Table S1.

#### Experimental images at different focal planes

Experimental images at different focal positions are collected from the open-source Parasite Datasets provided by Zhang et al. [22] and Jiang [23]. We have selected 11 confocal measurements of BPAE cell nuclei stained with blue-fluorescent DNA stain. The images are acquired by a high-resolution confocal scanning microscope with a size of 1,024 × 1,024 pixels at 7 focal positions along the *z* direction of the optical axis: at the optimal focal plane and at steps of 0.6, 1.2, and 1.8 μm along the *z* axis, below and above the ideal focus. These images are utilized to demonstrate the applicability of MMSim for defocusing problems and to prove its feasibility for RR implementations.

### Validation of metrics through correlation measures

The performance of MMSim against state-of-the-art metrics is evaluated by correlating each IQA method with the real image degradation through Pearson linear correlation coefficient, Kendall rank correlation coefficient, and root mean squared error. To compute this evaluation, we use measurements with known experimental artifacts, for which it is possible to sort the images objectively according to their quality. Depending on the strength of the artifact, we assign an integer number that represents a quality score. A score from 0 to 3 is assigned to out-of-focus images. A score of 0 is assigned to the positions furthest from the optimal focus, while a score of 3 is assigned to in-focus images. In the case of images exhibiting increasing noise (Fig. 4), we test 2 approaches: in the first one, a score from 1 to 6 is assigned to the images generated using an increasing number of averaged images, while in the second configuration, the score is the number of images averaged to obtain the measurement (1, 2, 4, 8, 16, and 50). Finally, these quality scores are correlated with the similarity predicted by each metric. The results are reported in Figs. S6 to S8.

## Data Availability

The source code for MMSim is publicly available in the GitLab repository https://git.photonicdata.science/elena.corbetta/multi-marker-similarity with a small demo dataset. The images used in this study are taken or generated from open-source datasets. Source images and source data for the reproduction of the results of this study are available from the authors upon reasonable request.
