# Supplementary material for: Multi-marker Similarity Enables Reduced-Reference and Interpretable Image Quality Assessment in Optical Microscopy
Source: Research (Wash D C). 2025 Jul 18;8:0783. doi: 10.34133/research.0783 (PMC12271742; doi:10.34133/research.0783)
Supplement: Supplementary 1 — Figs. S1 to S8 Table S1 [file research.0783.f1.pdf]

Research
A SCIENCE PARTNER JOURNAL
SPJ
SCIENCE PARTNER JOURNALS

Multi-Marker Similarity Enables Reduced-Reference and Interpretable Image Quality Assessment in Optical Microscopy.

Elena Corbetta[1,2*], Thomas Bocklitz[1,2*]

[1]Leibniz Institute of Photonic Technology, Member of Leibniz Health Technologies, Member of the Leibniz Centre for Photonics in Infection Research (LPI), 07745 Jena, Germany.
[2]Institute of Physical Chemistry (IPC) and Abbe Center of Photonics (ACP), Friedrich Schiller University Jena, Member of the Leibniz Centre for Photonics in Infection Research (LPI), 07743 Jena, Germany
[*]Address correspondence to: thomas.bocklitz@uni-jena.de

SUPPLEMENTARY MATERIALS

Overview of the images used to validate multi-marker similarity

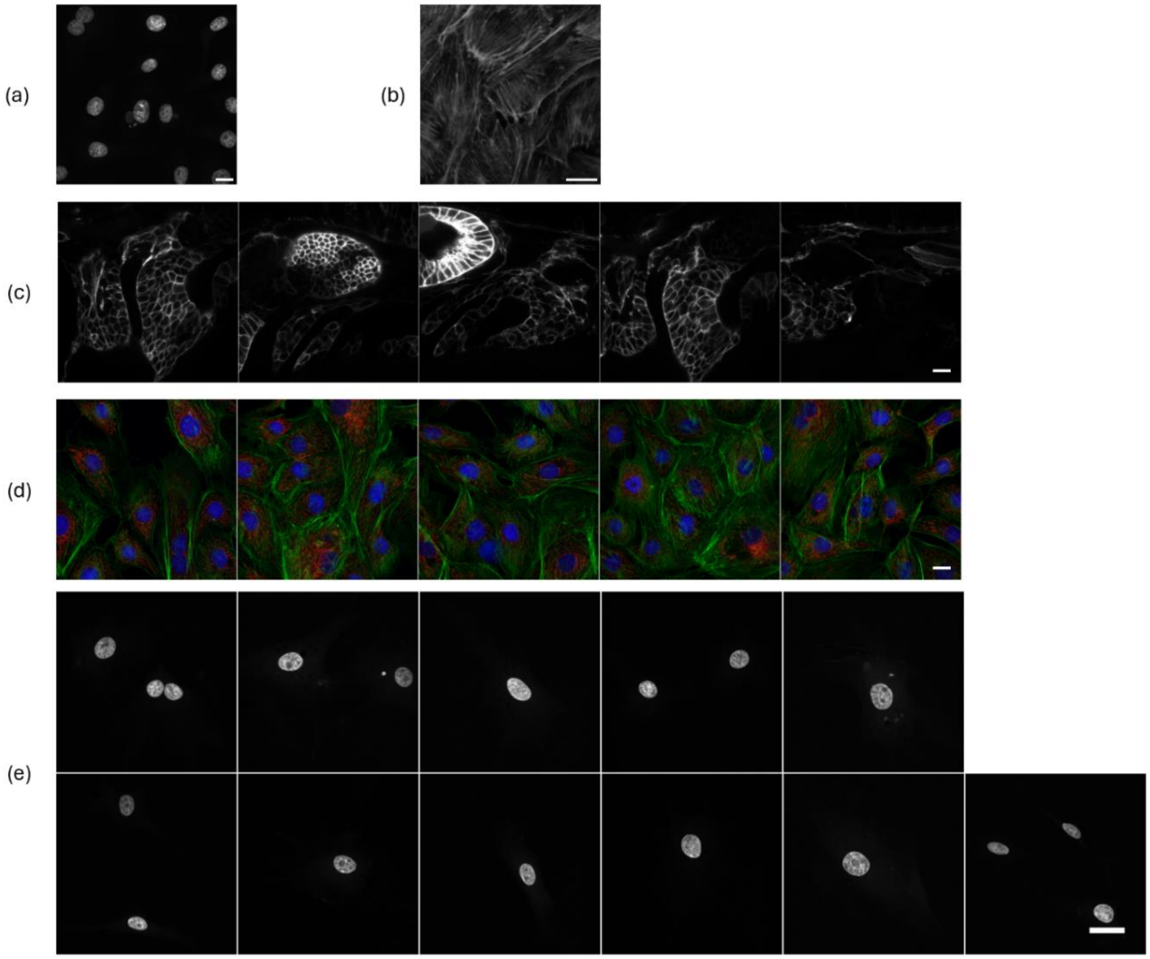

Fig. S1– ***Full-size high-quality images of the samples used in this study.*** *(a) Fluorescence confocal measurement of BPAE cells nuclei from the FMD dataset. Scale bar: 15 µm. (b) Widefield measurement of BPAE cells F-actin (green channel) from the FMD dataset. Scale bar: 15 µm. (c) Confocal fluorescence measurement of zebrafish embryos from the FMD dataset. Scale bar: 15 µm. (d) Fluorescence confocal measurement of BPAE cells with three channels measured in parallel from the FMD dataset: mitochondria (red), F-actin (green) and nuclei (blue). Scale bar: 15 µm. (e) Fluorescence confocal measurement of BPAE cells from the dataset by C. Zhang et al. Scale bar: 200 px.*

Comparison of multi-marker similarity (MMSim) with state-of-the-art metrics for Gaussian denoising

A high-quality confocal microscopy measurement of BPAE cells is degraded by mixed Poisson-Gaussian noise and then denoised by Gaussian filters with increasing standard deviation. Fig. S2 shows a detailed characterization of panels (d,e) of Fig. 1 of the main text. Fig. S2 (b) highlights the significant difference in evaluation between state-of-the-art methods. According to visual perception, the image selected as the best by PSNR and MSE appears too smooth. SSIM and MAE are also unreliable for this optimization problem. Compared to the Gaussian denoising shown in Fig. 2(b) later in the manuscript, this example starts with a higher level of noise, particularly in the background. Consequently, some state-of-the-art metrics may select highly smoothed images due to the large degree of similarity in the background region rather than evaluating the quality of the imaged sample. Conversely, MMSim selects an image that significantly removes the simulated noise while preserving the sharp features of the cell nuclei.

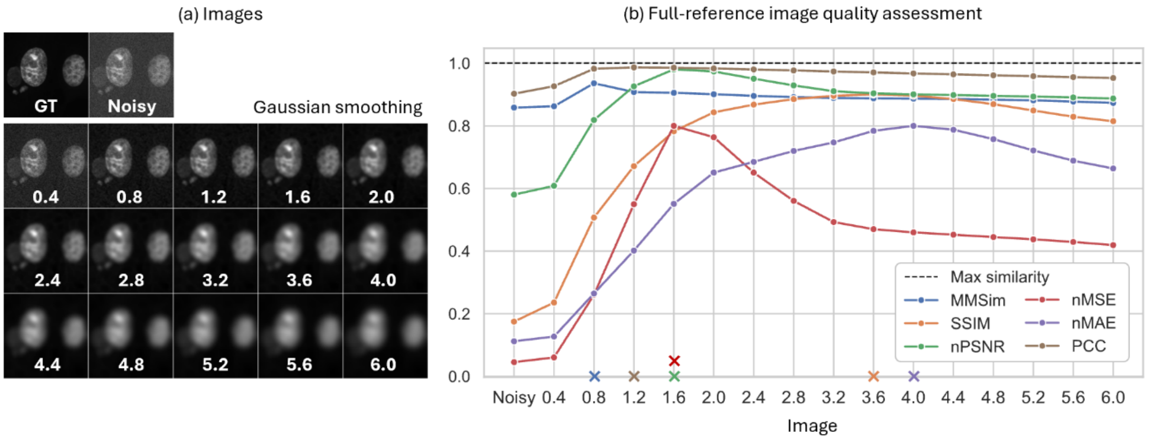

*Fig. S2 - **Image quality assessment (IQA) for fluorescence microscopy images denoised by Gaussian smoothing.** (a) Crops of the images used for this example. GT is the reference image, Noisy is the image with mixed Poisson-Gaussian noise, and the following images are denoised by Gaussian filtering with standard deviation (in pixel) as reported in the labels. (b) MMSim compared with five state-of-the-art metrics: structural similarity index (SSIM), peak signal-to-noise ratio (PSNR), mean squared error (MSE), mean absolute error (MAE), Pearson's correlation coefficient (PCC). nPSNR, nMSE and nMAE are normalized to be bound to 0-1 for a comparable visualization with the other FR metrics. The colored crosses mark the image that scores the best value for each metric.*

Variability of single-marker similarity for different denoising methods

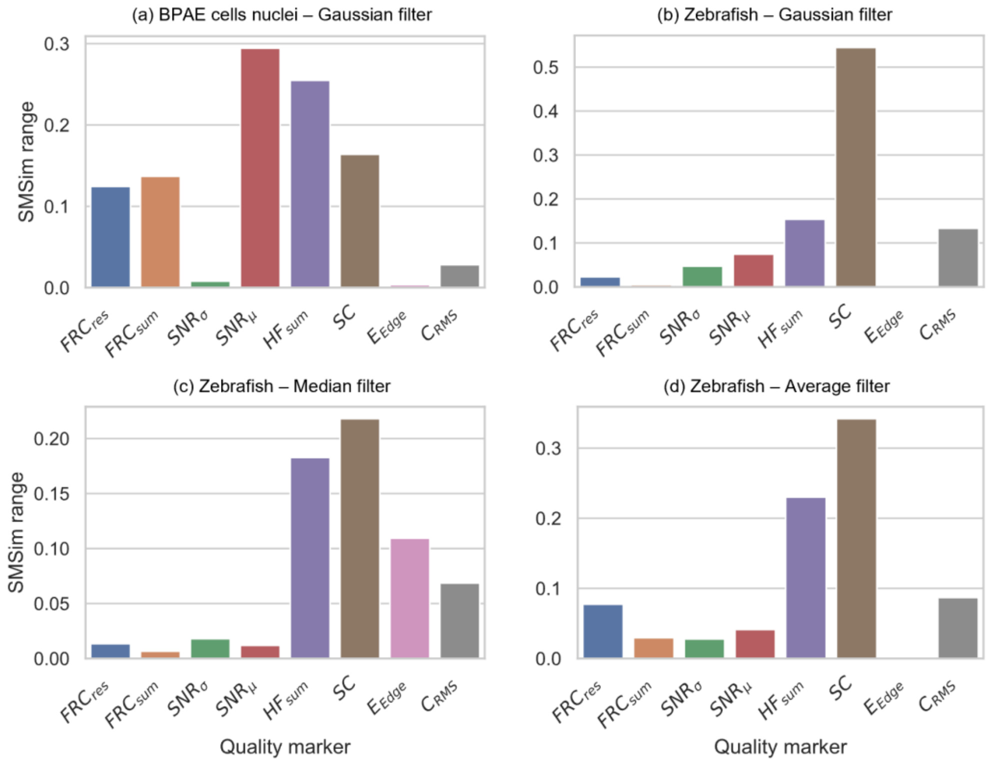

*Fig. S3 –**Variability of Single-Marker Similarity for sets of images of BPAE cells nuclei and zebrafish embryos denoised by different methods.** This variability is computed as the minimum to maximum range of the single-marker similarity score within the dataset. The relevance of different quality markers for detecting differences between images depends on the processing case. (a) A noisy semisynthetic image of BPAE cell nuclei is denoised using a Gaussian filter with an increasing standard deviation, as shown in Fig. 1. (b, c, d) Five noisy FOVs of zebrafish embryos are denoised, respectively, by a Gaussian filter, a median filter, and an average filter as shown in Fig. 3.*

Optimization of denoising results of multi-channel images

MMSim and state-of-the-art FR metrics are utilized to predict the best combination of denoising results for RGB images of BPAE cells. In Fig. S4, noisy and GT images of 5 different FOVs are compared to the optimized images selected by the quality metrics. Each channel is denoised and evaluated independently, then the best denoised channels for each FOV is predicted by the metrics, generating the multi-channel images by combining the best denoised image of each channel. In general, all the metrics show a good result, even if PCC is biased towards higher levels of smoothing. MMSim recovers well the image features and selects images with similar quality, independently on the FOV. PSNR, MSE and MAE show also a stable performance. SSIM selects images with good quality, with a bias towards stronger smoothing for the green and blues channel.

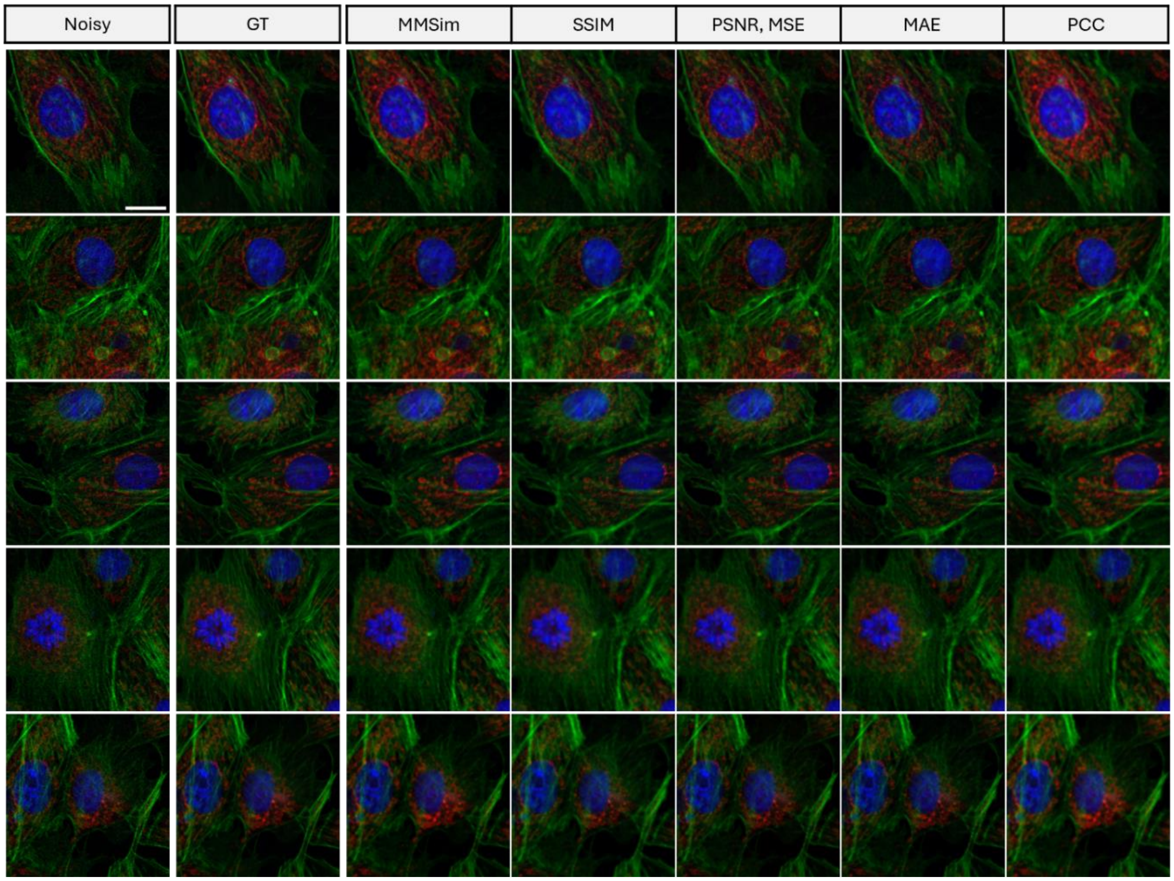

*Fig. S4 – **Optimization of 3-channel measurements of BPAE cells.** Noisy images are denoised by 5 denoising methods (average filter, Gaussian filter, median filter, total variation regularization, and wavelet filtering), applied with 5 values of the hyperparameters, as in Fig. 3 of the main manuscript. The plot shows the GT images, obtained by averaging 50 noisy measurements, and the optimized combination of denoised channels as predicted by MMSim and other FR metrics. All metrics show a good performance, except for PCC, that selects images with excessive smoothing. MMSim shows a good recovery of all channels, selecting images with a similar quality across different measurements. SSIM selects more smoothed images for the green and blue channels and weaker smoothing for red channel. Scale bar: 15 µm.*

Global quality ranking for measurements of 20 FOVs of F-actin in BPAE cells with increasing noise levels

Here, we demonstrate that MMSim can detect small degradations in high-quality images. It does so by identifying decreases in darkness and less structured regions within fields of view (FOVs) that are marked as reference images. For this purpose, an additional RR similarity estimation is generated for wide-field BPAE cell (F-actin channel) measurements of 20 FOVs taken from the FMD dataset. For each FOV, six noise levels are generated by averaging 1, 2, 4, 8, 16 and 50 images. FOV 18, with 50 averaged images, was selected as the ground truth because it contains a variety of structures, sharp features, and bright signal. The global quality rankings generated by different metrics are shown in Fig. S, colored according to the FOV (a) and the number of averaged images (b). As stated in the main manuscript, state-of-the-art metrics exhibit FOV-dependent behavior and are less dependent on noise levels than MMSim. Our method can extract quality markers and generate global rankings in which the top images are selected based on real quality.
Upon closer inspection, we observe that a few images with low noise content (dark blue in panel (b)) are ranked lower in the MMSim ranking. Panel (c) provides a closer look at these images and shows that they are FOVs from darker or blurrier acquisitions. The high visual similarity between the ground truth (GT) and image 1 is not comparable to the visual similarity between images 34, 36, and 40. Conversely, image 16, which is ranked higher despite its higher noise level, shows sharp features and good brightness.

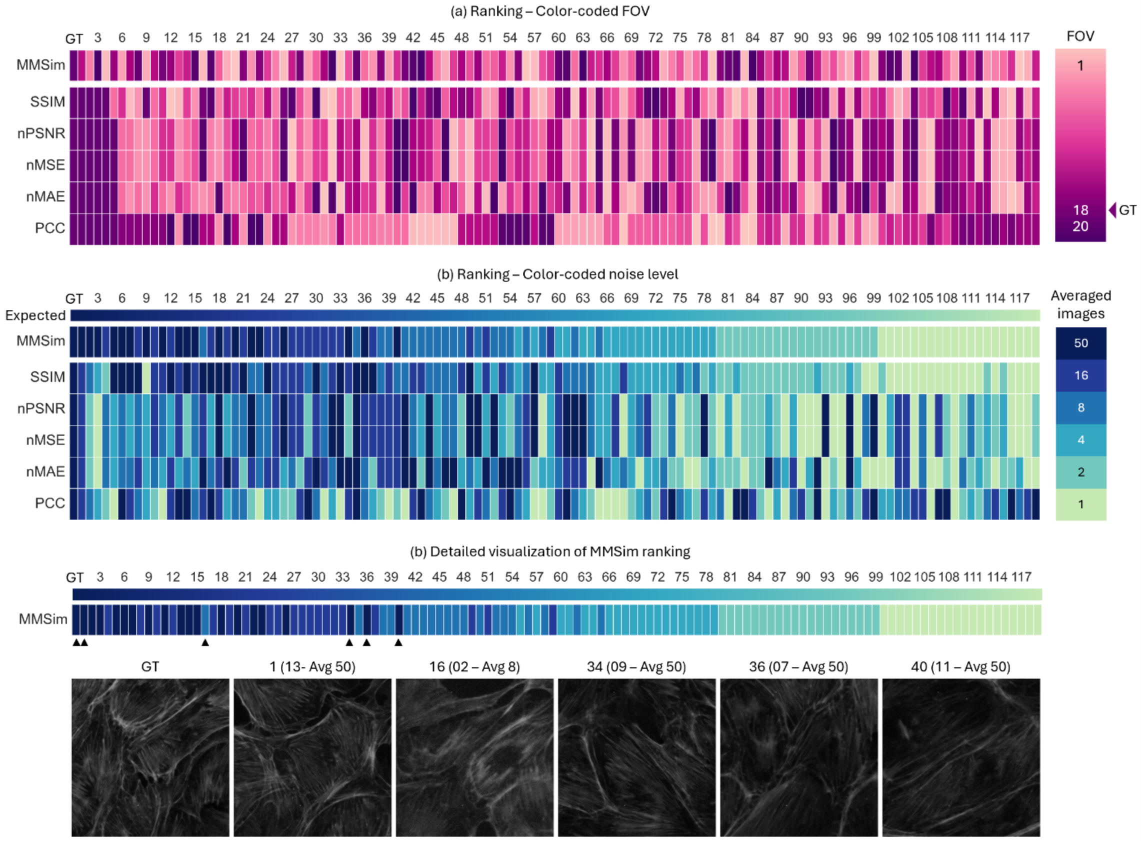

*Fig. S5 - **Global quality ranking for BPAE cell F-actin widefield measurements at increasing noise levels (20 FOVs).** Each row in the plots contains the ranking generated by a different metric in a RR configuration, with FOV 18 selected as the ground truth (GT). The same ranking is represented by assigning different colors to the FOV (panel (a)) and to the noise level (panel (b)). A colored bar at the top of panel (b) (Expected) shows the*

expected color distribution of the images in the ranking. For an ideal evaluation, the result should depend only on image quality, with shuffled FOVs and a high number of averaged images in the top positions. (a) Color-coded FOV. The MSSim ranking is independent of the FOV; the SSIM ranking is weakly dependent on the FOV; and the other rankings are strongly dependent on the FOV. (b) The same quality ranking as in (a), but the color indicates the number of averaged images. (c) A detailed visualization of some images from the MMSim ranking is shown by the black arrowheads to visually inspect their quality.

Correlation between real degradation level and FR metrics

The performance of MMSim is validated against the state-of-the-art metrics. We computed the Pearson linear correlation coefficient (PLCC), the Kendall rank correlation coefficient (KRCC), and the root mean squared error (RMSE) for all the full-reference (FR) metrics. The metrics are correlated with objective scores assigned to the images according to the real experimental degradation. The scores have higher values for increasing image quality. A good result is obtained by maximizing PLCC and KRCC results and by minimizing the RMSE.
Fig. S6 shows the correlation results for the confocal measurements of fixed zebrafish embryos with increasing noise levels. The correlation is computed in a FR configuration, selecting one GT for each field of view, and it is repeated for two different objective scores: in the first case, the scores are increasing integer numbers from 0 to 6 (Fig. S6 (a)), while in the second case they correspond to the number of images averaged to obtain each noise level (Fig. S (b)). In this case, our method shows good correlation results, reaching values of PLCC, KRCC and RMSE very close to the best ones. MMSim shows poor correlation only for PLCC in panel (b); nevertheless, it reaches good values of KRCC and RMSE in the same panel, demonstrating that it is comparable or better than established state-of-the-art approaches.

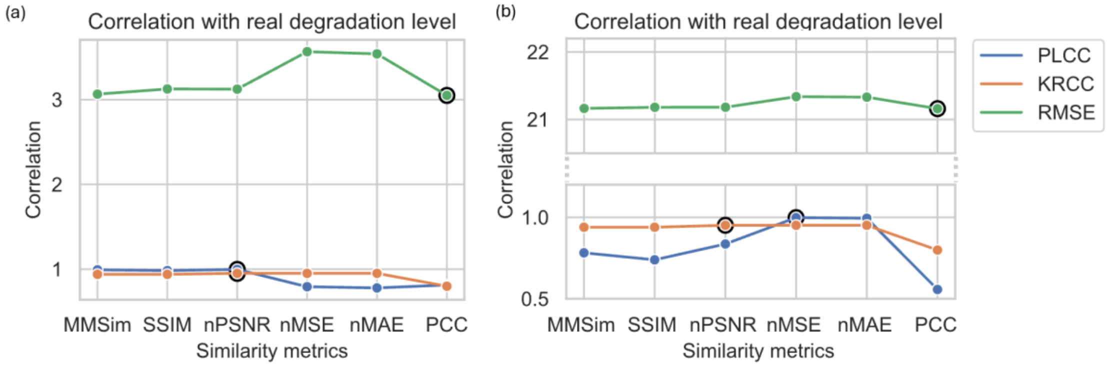

Similarity metrics	PLCC	KRCC	RMSE
MMSim	0,992	0,939	3,065
SSIM	0,983	0,939	3,126
nPSNR	0,997	0,951	3,124
nMSE	0,793	0,951	3,566
nMAE	0,779	0,951	3,540
PCC	0,813	0,799	3,050

Similarity metrics	PLCC	KRCC	RMSE
MMSim	0,783	0,939	21,164
SSIM	0,739	0,939	21,180
nPSNR	0,836	0,951	21,181
nMSE	0,998	0,951	21,338
nMAE	0,994	0,951	21,331
PCC	0,557	0,799	21,160

Fig. S6 – ***Correlation measures computed for five confocal measurements of fixed zebrafish embryos with different noise levels.*** *Similarity metrics computed in a FR configuration. (a) The score assigned to the images is an integer number from 0 (raw image) to 6 (high-quality image), increasing with the image quality. (b) The score corresponds to the number of images averaged to obtain each noise level. They are, respectively, from the raw image to the high-quality image: 1, 2, 4, 8, 16, 50. The black round frames in the plots indicate the best correlation result among the metrics.*

Fig. S shows the correlation results for the same images, but in the reduced-reference (RR) configuration, as reported in the main manuscript (Fig. 4). MMSim confirms its feasibility for RR evaluations, reaching the best correlation results for PLCC and KRCC, and minimizing the RMSE. Fig. S8 shows the correlation results obtained for images at different focal positions in a reduced reference configuration (Fig. 6). In both cases, MMSim shows

the best result, demonstrating the reliability of the method and its ability to track the changes induced by the image degradations by following the changes in the quality markers. In both cases, the evaluation is executed in a reduced-reference configuration.

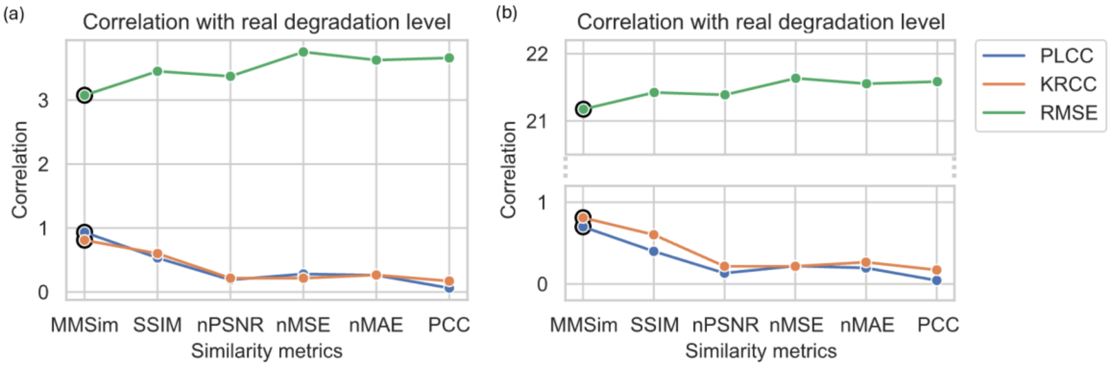

Similarity metrics	PLCC	KRCC	RMSE
**MMSim**	0,926	0,785	3,078
**SSIM**	0,533	0,602	3,451
**nPSNR**	0,190	0,216	3,373
**nMSE**	0,281	0,216	3,753
**nMAE**	0,262	0,266	3,626
**PCC**	0,062	0,171	3,660

Similarity metrics	PLCC	KRCC	RMSE
**MMSim**	0,695	0,785	21,176
**SSIM**	0,399	0,602	21,424
**nPSNR**	0,132	0,216	21,389
**nMSE**	0,220	0,216	21,635
**nMAE**	0,196	0,266	21,554
**PCC**	0,043	0,171	21,585

Fig. S7 – ***Correlation measures computed for five confocal measurements of fixed zebrafish embryos with different noise levels.*** *Similarity metrics computed in a RR configuration. (a) The score assigned to the images is an integer number from 0 (raw image) to 6 (high-quality image), increasing with the image quality. (b) The score corresponds to the number of images averaged to obtain each noise level. They are, respectively, from the raw image to the high-quality image: 1, 2, 4, 8, 16, 50. The black round frames in the plots indicate the best correlation result among the metrics, obtained always by MMSim.*

Similarity metrics	PLCC	KRCC	RMSE
**MMSim**	0,847	0,746	1,033
**SSIM**	0,762	0,622	1,180
**nPSNR**	0,677	0,526	1,061
**nMSE**	0,598	0,526	1,256
**nMAE**	0,704	0,619	1,257
**PCC**	0,086	0,029	1,492

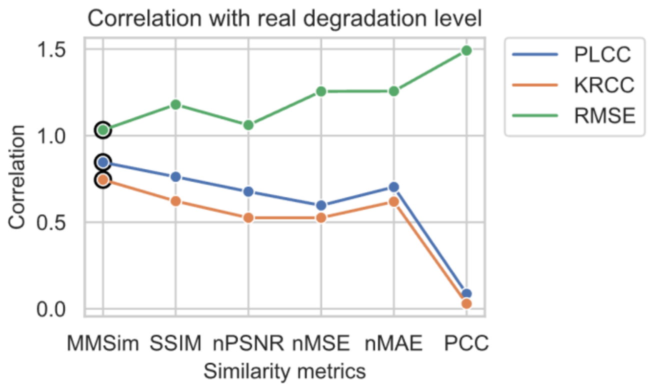

Fig. S8 – ***Correlation measures computed for eleven confocal measurements of BPAE cells at seven different focal positions along the optical axis.*** *Similarity metrics computed in a FR configuration. The score assigned to the images is an integer number from 0 (strongest defocusing) to 3 (optimal focal plane). The black round frames in the plots indicate the best correlation result among the metrics, obtained always by MMSim.*

Supplementary Table 1

Denoising method	Hyperparameter	P1	P2	P3	P4	P5
Average filter	Lateral filter size / px	3	5	7	9	11
Gaussian filter	Standard deviation of 2D Gaussian function / px	1	2	3	4	5
Median filter	Lateral filter size / px	3	5	7	9	11
Total Variation regularization	Denoising weight	0.01	0.1	0.5	1	2
Wavelet filtering (db1)	Noise standard deviation	10	20	30	40	50

Table S1 – ***Denoising methods and relative hyperparameters utilized to evaluate experimental denoised images.***
